# Natural variation in cold and heat survival among temperate and tropical *Caenorhabditis briggsae*

**DOI:** 10.17912/micropub.biology.001645

**Published:** 2025-06-25

**Authors:** Paul Vigne, Christian Braendle

**Affiliations:** 1 Université Côte d’Azur, CNRS, Inserm, IBV, Nice, France

## Abstract

We developed a simple assay to quantify natural variation in adult thermal stress tolerance in the nematode *Caenorhabditis briggsae* . Selfing hermaphrodites from wild strains were exposed to cold (0°C, 5°C) or heat (29°C, 33°C, 35°C) for 15 hours during early adulthood. Cold exposure revealed clear clade-level differences: temperate strains showed high survival, while tropical strains were more variable and generally less tolerant. Heat exposure at 35°C caused high mortality across strains from both clades, with only slightly lower mortality in tropical strains. At 29°C, all strains showed full survival. By 33°C, mortality rose modestly, with temperate strains showing slightly greater sensitivity. This assay captures natural variation in thermal tolerance and provides a simple and efficient tool for studying thermal adaptation in *C. briggsae* and other *Caenorhabditis* nematodes.

**Figure 1. Adult survival of C. briggsae wild strains following exposure to extreme temperatures:**
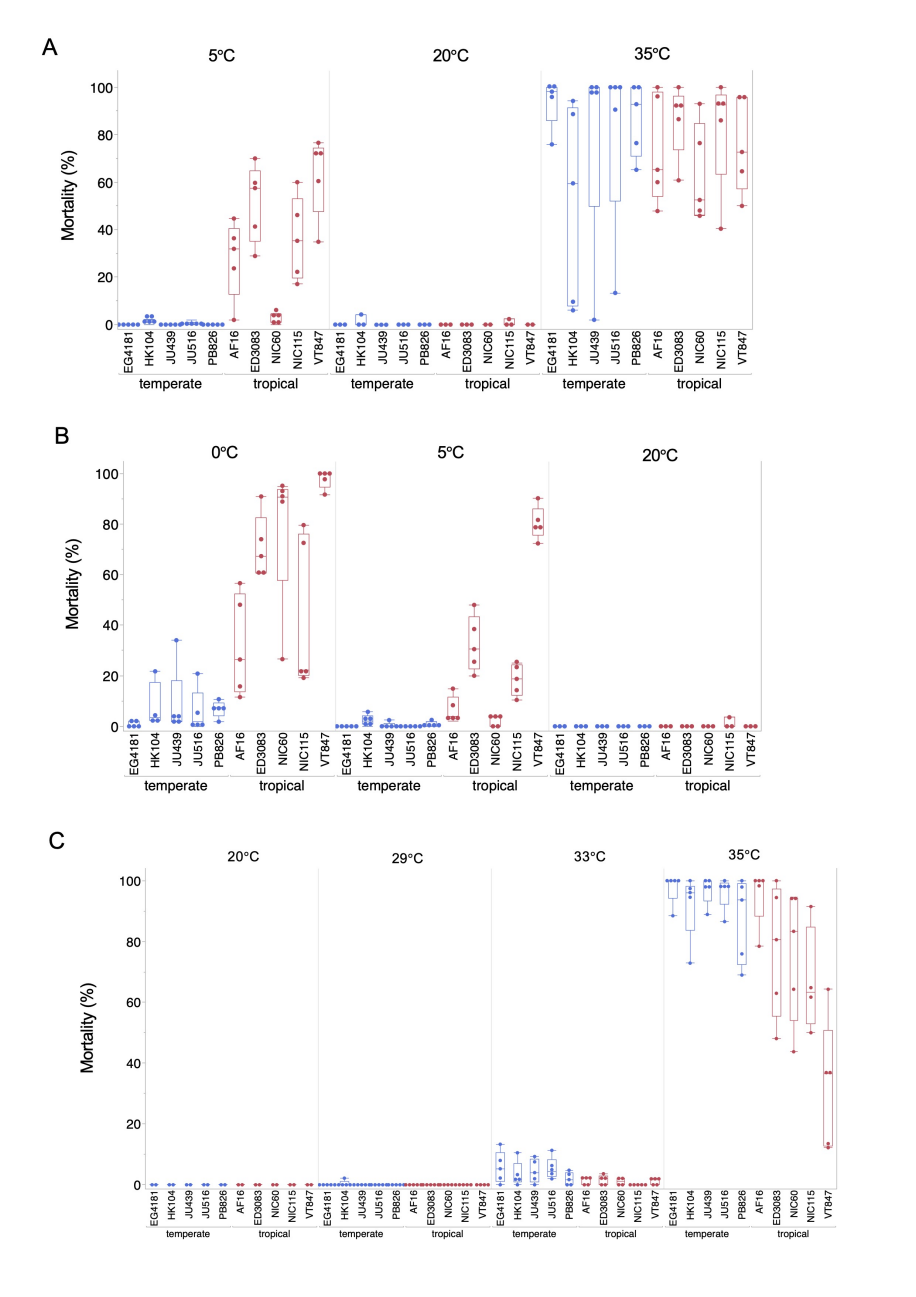
Replicated populations of adult hermaphrodite (L4 + 24h) were exposed to acute cold (0°C, 5°C) or heat (29°C, 33°C, 35°C) stress for 15 hours. Following a 24-hour recovery period at 20°C, the proportion of dead individuals was scored. We used quasi-binomial generalized linear models (GLMs) to assess the effects of clade and strain (nested within clade) on mortality in treatment conditions that caused significant mortality. **(A)** Survival following exposure to 5°C, 20°C (control), and 35°C. At 5°C, clade had a strong effect on mortality: tropical strains exhibited significantly higher mortality than temperate strains (β = 4.96, SE = 0.45, z = 10.97, p < 0.0001; dispersion = 0.16). Strain variation was significant within the tropical clade, with several strains showing markedly elevated mortality, whereas temperate strains exhibited almost no mortality. At 35°C, the effect of clade was not statistically significant (β = –0.13, SE = 0.10, z = –1.34, p = 0.18; dispersion = 0.45); however, statistically significant differences were observed among strains within each clade. **(B)** Survival following exposure to 0°C, 5°C, and 20°C (control). At 0°C, tropical strains exhibited significantly higher mortality than temperate strains (β = 3.39, SE = 0.14, z = 24.57, p < 0.0001, dispersion = 0.29). Similar patterns were observed at 5°C, with the clade effect remaining highly significant (β = 4.07, SE = 0.36, z = 11.29, p < 0.0001, dispersion = 0.23). Statistically significant differences were observed among strains within tropical clades at both temperatures, and at 0°C for strains within the temperate clade. **(C)** Survival following exposure to 20 °C (control), 29°C, 33°C, and 35°C. At 33°C, tropical strains had significantly lower mortality than temperate strains (β = –1.37, SE = 0.32, z = –4.22, p < 0.0001; dispersion = 0.026); strain variation within the temperate clade was highly significant. At 35°C, tropical strains maintained lower mortality than temperate strains (β = –1.77, SE = 0.13, z = –13.48, p < 0.0001; dispersion = 0.25). Strain effects within both clades were statistically significant.

## Description

Temperature is a central factor affecting organismal physiology, especially in ectotherms, where it governs metabolic rates, development, and reproduction (Angilletta et al. 2002). It thus plays a key role in shaping species distributions, population dynamics, and evolutionary processes (Huey and Kingsolver 1989). Many ectotherms exhibit substantial intraspecific variation in thermal tolerance, providing a basis for local adaptation (Sunday et al. 2019). Studying these responses can reveal the genetic and physiological mechanisms underlying thermal resilience and vulnerability.

The androdioecious (hermaphrodite–male) nematode *Caenorhabditis briggsae* is a globally distributed species, with a subset of genetically divergent natural strains that can be grouped into tropical and temperate phylogeographic clades (Cutter et al. 2006; Félix et al. 2013; Thomas et al. 2015). These clades exhibit clear signatures of local thermal adaptation, as evidenced by consistent differences in thermal performance traits—such as fecundity and behavior—when exposed to sustained temperature variation (Prasad et al. 2011; Poullet et al. 2015; Wang et al. 2021). Moreover, substantial variation in thermal responses exists not only between the temperate and tropical clades but also within each clade, and strains that do not cluster within either major phylogeographic group also exhibit diverse thermal phenotypes, suggesting that adaptation to local thermal environments is complex and has likely arisen independently across multiple evolutionary lineages (Cutter et al. 2006; Prasad et al. 2011; Baird and Stonesifer 2012; Félix et al. 2013; Stegeman et al. 2013, 2019; Crombie et al. 2019; Wang et al. 2021; Jhaveri and Andersen 2025; Jhaveri et al. 2025). While heat tolerance has been relatively well-characterized in *Caenorhabditis briggsae* hermaphrodites, less is known about responses to cold exposure. In one study (Wang et al. 2021), strains reared at 20°C and then exposed to 4°C for 60 hours showed striking differences: temperate strains fully recovered, while tropical strains experienced high mortality. Additional strains outside the canonical temperate and tropical clades followed similar patterns—those from colder regions like Quebec (Canada) were cold-tolerant, while early-diverging tropical strains such as those from Kerala (India) were not—suggesting that cold resistance is a derived trait in lineages from cooler climates (Wang et al. 2021).

Building on previous studies, we developed a simplified, rapid assay to evaluate thermal tolerance in *C. briggsae* , using a standardized 15-hour exposure to assess both cold and heat tolerance in parallel. This approach enables efficient phenotyping and direct comparisons across thermal conditions. The 15-hour duration was selected after pilot experiments showed that shorter exposures (10–12 h) yielded minimal mortality and poor strain discrimination, while longer exposures caused near-complete lethality. The 15-hour period thus provided the most reproducible and discriminatory compromise, while also reflecting transient natural events such as overnight cold exposure.

We first assessed (self-fertilizing) hermaphrodite adult survival (L4 + 24 h) following a 15-hour exposure to cold (5°C) or heat (35°C). Overall, we observed significant natural variation in survival ( Fig. 1A ), with clearest differences seen after cold exposure. Tropical strains generally exhibited higher mortality, though survival varied widely among them. In contrast, temperate strains showed almost no mortality when exposed to 5°C. After heat exposure, mortality was higher across all strains but also highly variable ( Fig. 1A ). We then asked whether a more severe cold exposure (0°C) would induce mortality in temperate strains. As expected, temperate strains exhibited low but consistent mortality at 0°C, while tropical strains showed increased mortality compared to 5°C ( Fig. 1B ). Finally, we tested whether milder heat exposures (29°C and 33°C) could uncover strain differences. At 29°C, no mortality was observed in either tropical or temperate strains ( Fig. 1C ). At 33°C, both clades exhibited low but consistent levels of mortality, with a trend toward slightly higher mortality in temperate strains ( Fig. 1C ). These results indicate that while 29°C did not reduce survival in *C. briggsae* adults, exposure to 33°C begins to cause mortality, with clade-level differences in thermal sensitivity emerging at this threshold. We also observed substantial within-strain variation in survival at 35 °C ( Fig. 1A and 1C), with some replicates of the same strain showing mortality as low as ~10% and others exceeding 80%. This variability may reflect the proximity of 35 °C to the upper thermal tolerance threshold, where small environmental or biological fluctuations can cause disproportionate effects on survival. Possible contributing factors include stochastic physiological responses near a critical temperature, as well as incubator fluctuations, which could shift actual exposure conditions. Minor differences in nematode density, bacterial lawn condition, or microclimate of individual plates may also contribute to this sensitivity.

Taken together, our results demonstrate significant natural variation in *C. briggsae* thermotolerance, confirming consistent clade-level differences in cold tolerance (Wang et al. 2021), with temperate strains showing high survival at both 0 °C and 5°C, and tropical strains displaying greater sensitivity and broader variation. In contrast, exposure to 35°C led to high mortality across all strains, and only mild, less variable effects were observed at 33°C, particularly in temperate strains. This asymmetry could suggest that lower thermal limits may reveal more genetic variation than upper limits. Our standardized 15-hour assay provides a simple and effective tool for simultaneously assessing natural variation in responses to cold and heat stress in *Caenorhabditis* nematodes.

## Methods

Strains, nematode culture and age synchronization

We used five wild isolates from each clade, with strains assigned to either the temperate clade ( EG4181 , HK104 , JU439 , JU516 , PB826 ) or the tropical clade ( AF16 , ED3083 , NIC115 , NIC60 , VT847 ), as previously defined (Cutter et al. 2006).

Populations were synchronized by bleaching gravid adults to strain embryos, which were allowed to hatch overnight in M9 buffer to obtain synchronized L1 larvae. These L1 larvae were transferred to 5.5 cm NGM (nematode growth medium) agar plates (2.5% agar) seeded with a lawn of *E. coli* OP50 and incubated at 20°C (Stiernagle 2006). Worms were allowed to grow until the young adult stage, defined as 24 hours post-L4 molt (L4+24h).

Each of the three panels in Figure 1 (A–C) represents a distinct experiment in which all temperatures and strains were tested in parallel, using animals derived from the same cohort. All strains were maintained under identical conditions (20°C with ad libitum food) for three generations prior to the assay.

Temperature treatments

For each strain-temperature combination, an average of 4 replicates (NGM plates) were prepared, with a mean of 50 animals per replicate (see Extended Data for exact numbers). Young adult hermaphrodites were gently transferred using a platinum wire pick to fresh NGM plates seeded with OP50 and then immediately incubated at the designated temperatures for 15 hours. After exposure to treatment and control conditions, animals were transferred to 20°C to recover.

Experiments were conducted using MIR-254-PE cooled incubators (PHCbi), which maintain temperatures with a variation of up to ±1.5°C. Temperature precision is reduced at low temperatures due to compressor cycling.

Scoring of adult survival

Survival was assessed 24 hours after the return to 20°C. All animals were gently prodded with a platinum wire, and individuals that responded with movement were scored as alive. Immobile animals showing no response were scored as dead. Worms with desiccated or ruptured cuticles were excluded from analysis. All survival assays were performed on the same batch of NGM plates under consistent humidity and environmental conditions to minimize batch effects.

Statistical analysis

We used quasi-binomial generalized linear models (GLMs) to analyze mortality, defined as the proportion of dead individuals per replicate, as a function of clade and strain (nested within clade). Analyses were conducted separately for each temperature condition (e.g., 5°C and 35°C). Models were specified with a logit link function and weighted by the number of individuals per replicate. To account for overdispersion, we estimated the dispersion parameter as the ratio of residual deviance to residual degrees of freedom and adjusted standard errors accordingly. Clade effects were assessed by fitting models with clade as the sole fixed effect. To evaluate strain effects, additional models were fit separately within each clade, treating strain as a categorical fixed factor. All analyses were performed in Python using the *statsmodels* package (v0.14.4).

## Data Availability

Description: Complete raw data of adult survival of C. briggsae. Resource Type: Dataset. DOI: https://doi.org/10.22002/8zkxy-4k883

## References

[R1] Angilletta MJ Jr, Cooper BS, Schuler MS, Boyles JG (2010). The evolution of thermal physiology in endotherms.. Front Biosci (Elite Ed).

[R2] Baird SE, Stonesifer R (2012). Reproductive isolation in Caenorhabditis briggsae: Dysgenic interactions between maternal- and zygotic-effect loci result in a delayed development phenotype.. Worm.

[R3] Crombie TA, Zdraljevic S, Cook DE, Tanny RE, Brady SC, Wang Y, Evans KS, Hahnel S, Lee D, Rodriguez BC, Zhang G, van der Zwagg J, Kiontke K, Andersen EC (2019). Deep sampling of Hawaiian
*Caenorhabditis elegans*
reveals high genetic diversity and admixture with global populations.. Elife.

[R4] Cutter AD, Félix MA, Barrière A, Charlesworth D (2006). Patterns of nucleotide polymorphism distinguish temperate and tropical wild isolates of Caenorhabditis briggsae.. Genetics.

[R5] Félix MA, Jovelin R, Ferrari C, Han S, Cho YR, Andersen EC, Cutter AD, Braendle C (2013). Species richness, distribution and genetic diversity of Caenorhabditis nematodes in a remote tropical rainforest.. BMC Evol Biol.

[R6] Huey RB, Kingsolver JG (1989). Evolution of thermal sensitivity of ectotherm performance.. Trends Ecol Evol.

[R7] Jhaveri NS, Andersen EC (2025). Fecundities of Hawaiian Caenorhabditis briggsae wild strains are not correlated with natural niche temperatures.. MicroPubl Biol.

[R8] Jhaveri N, Bhullar H, Sternberg PW, Gupta BP (2025). Heat tolerance and genetic adaptations in Caenorhabditis briggsae: insights from comparative studies with Caenorhabditis elegans.. Genetics.

[R9] Poullet N, Vielle A, Gimond C, Ferrari C, Braendle C (2015). Evolutionarily divergent thermal sensitivity of germline development and fertility in hermaphroditic Caenorhabditis nematodes.. Evol Dev.

[R10] Prasad A, Croydon-Sugarman MJ, Murray RL, Cutter AD (2010). Temperature-dependent fecundity associates with latitude in Caenorhabditis briggsae.. Evolution.

[R11] Stegeman GW, de Mesquita MB, Ryu WS, Cutter AD (2012). Temperature-dependent behaviours are genetically variable in the nematode Caenorhabditis briggsae.. J Exp Biol.

[R12] Stegeman GW, Baird SE, Ryu WS, Cutter AD (2019). Genetically Distinct Behavioral Modules Underlie Natural Variation in Thermal Performance Curves.. G3 (Bethesda).

[R13] Stiernagle T (2006). Maintenance of C. elegans.. WormBook.

[R14] Sunday J, Bennett JM, Calosi P, Clusella-Trullas S, Gravel S, Hargreaves AL, Leiva FP, Verberk WCEP, Olalla-Tárraga MÁ, Morales-Castilla I (2019). Thermal tolerance patterns across latitude and elevation.. Philos Trans R Soc Lond B Biol Sci.

[R15] Thomas CG, Wang W, Jovelin R, Ghosh R, Lomasko T, Trinh Q, Kruglyak L, Stein LD, Cutter AD (2015). Full-genome evolutionary histories of selfing, splitting, and selection in Caenorhabditis.. Genome Res.

[R16] Wang W, Flury AG, Garrison JL, Brem RB (2021). Cold Survival and Its Molecular Mechanisms in a Locally Adapted Nematode Population.. Genome Biol Evol.

